# How Painful are Lumbar Hernias? A Comprehensive Review of Intervention Strategies

**DOI:** 10.1007/s11916-024-01342-3

**Published:** 2025-01-25

**Authors:** Rafael Moreno-Gómez-Toledano, Irene Méndez-Mesón, Soledad Aguado-Henche, Alba Sebastián-Martín, Mónica Grande-Alonso

**Affiliations:** 1 Universidad de Alcalá, School of Medicine and Health Sciences, Department of Surgery, Medical and Social Sciences, Area of Human Anatomy and Embryology, Universidad de Alcalá, University Campus - C/ 19 Av de Madrid Km 33 600, 28871 Alcalá de Henares, Madrid Spain; 2 Departamento de Traumatología, Unidad de Columna, Hospital Universitario de Torrejón, Madrid, Spain; 3 Health Research Institute of Castilla-La Mancha (IDISCAM), Toledo, Spain; 4 Grupo de Investigación Clínico-Docente Sobre Ciencias de La Rehabilitación (INDOCLIN), Centro Superior de Estudios Universitarios La Salle, Madrid, Spain

**Keywords:** Lumbar hernia, Physiotherapy, Surgery, Pharmacology, Therapeutic exercise, Manual therapy

## Abstract

**Purpose of Review:**

Low back pain (LBP) is considered an important issue of public health, with annual prevalence estimations almost achieving 60% of the worldwide population. Available treatments have a limited impact on this condition, although they allow to alleviate pain and recover the patient’s quality of life. This review aims to go deeper on the understanding of this condition, providing an updated, brief, and concise whole picture of this common musculoskeletal problem.

**Recent Findings:**

Scientific literature, current clinical practice and clinical guidelines are summarized, focusing on three key aspects: classification of LBP, diagnosis of symptomatic lumbar hernia, and intervention strategies (conservative, surgical, and pharmacological). Benefits and drawbacks of each approach are tackled.

**Summary:**

The most appropriate intervention for LBP suffers is hitherto a conservative treatment based on therapeutic exercise, manual therapy and therapeutic education on the neurophysiological mechanisms of pain. Whether patient's condition is severe, does not improve with conservative treatment, or presents neurological symptoms, then surgical intervention is recommended. The efficiency of pharmaceutical approaches for LBP lacks high-quality evidence-based studies, and still needs to be in-depth explored. Current treatments help to improve symptoms and patient’s perspectives. However, further research in the field of herniated discs is essential in order to seek a therapy that could definitely cure or eliminate this condition.

## Introduction

Low back pain (LBP) is the most common musculoskeletal problem. Estimations show that the annual prevalence of LBP ranges from 18.6% to 57.4%, worldwide. [1, 2] Although it is not a high-risk pathology, it is considered the third leading cause of disability, only below ischaemic heart disease and chronic obstructive pulmonary disease. [3] Moreover, LBP is the most frequent occupational disorder due to its multifactorial nature and high chronicity, which makes it one of the main causes of absenteeism and medical consultations, with a considerable socio-economic cost. [3–5].

**Fig. 1:**
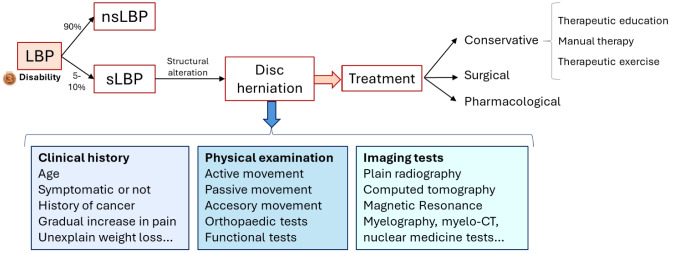
Schematic classification of low back pain. The representation includes a summary of the evaluation and treatment of disc herniation, a specific way of LBP involving structural alterations

It is relevant to note that, although in most cases acute LBP symptoms disappear within four to six weeks, this condition has a high rate of chronicity, which complicates its treatment. [6–8] LBP can be classified as specific (sLBP) or non-specific (nsLBP) (Fig. 1). sLBP is due to a structural alteration, although it is sometimes difficult to correlate the patient's symptoms with a specific source. [9] The prevalence of sLBP is estimated to be 5–10% with the most common diagnoses being disc herniation (5–10% of total sLBP) and spondylolysis (10%). [10, 11] On the other hand, nsLBP is not associated with any obvious pathology or damage and accounts for 90% of cases according to the World Health Organisation. [12] With regard to sLBP, specifically herniated discs, we should point out that it is a pathology that involves the alteration of an intervertebral disc due to a tear of the fibrous ring and the expulsion of the nucleus pulposus, which can give rise to symptoms such as local and radicular pain, followed by an affectation of sensorimotor, affective and cognitive variables. [13] Research studies show that the prevalence of this symptomatic clinical picture ranges from 1.6% to 13.4%, being more frequent in men between 30 and 50 years old. Importantly, a meta-analysis in 2017 found that up to 67% of hernias were reabsorbed with conservative treatment alone. [14–16] Among the most important risk factors for the development of a symptomatic lumbar hernia are age, male gender, strenuous activity, and smoking. [17] Considering the pathophysiology and aetiology of the disease, it is highly relevant to conduct a clinical interview and a series of diagnostic tests to confirm the presence of this clinical condition. This will allow for the establishment of realistic goals and appropriate treatment plans.

## Diagnosis of Symptomatic Lumbar Hernia

Physical examination by health professionals such as doctors and physiotherapists is of great importance, together with the clinical history and imaging tests. This is key due to the fact that scientific evidence determines that the presence of this pathology in imaging tests does not have to be directly related to the presence of symptoms. [18].

The physical examination should be based on the performance of active, passive and accessory movements, orthopaedic tests and functional tests that assess sensorimotor, affective and cognitive variables related to their pain. Regarding the orthopaedic tests that should be performed in the hypothesis of discogenic pain due to lumbar hernia, *Hancock *et al*.,* suggest that the physical examination should initially be accompanied by the straight leg raising test as an initial screening and subsequently, at least three of the following four conditions should be positive: [1] localisation of the pain in the dermatome that coincides with the affected root; [2] sensory deficit in the territory innervated by the said root; [3] motor reflex deficit in the territory innervated by said root and finally, [4] motor weakness of the muscles innervated by said root [19]. On the other hand, other studies suggest that patients with symptomatic lumbar hernia also tend to have significant pinching or burning along the path of the affected root, and centralisation of symptoms with repeated extension gestures. [9, 20–23] For that reason, it is important to include in the physical examination the test of repeated movement in flexion and extension, as well as tests of neural mechanosensitivity related to the affected root in the imaging tests.

With regard to imaging tests, we must bear in mind that when requesting one of these tests, the first thing to differentiate is whether it is mechanical low back pain or not. [24] For this purpose, so-called red flags are signs that alert us to the need for urgent or emergent imaging tests. Some of these red flags include: pain in individuals under 18 years old, in individuals over 50 years old with personal history of cancer, gradual increase in pain, unexplained weight loss, persistent pain in bed or at rest, parenteral drug users, immunosuppressed individuals, history of urinary or skin infection, fever, previous tuberculosis, osteoporosis, corticosteroid treatment, acute urinary retention, or neurological abnormalities. [25].

In the case of LBP without alarm symptoms, it is not necessary to immediately request imaging tests. Plain radiography is the first test to request in vertebral chronic or subacute pain without alarm signs/symptoms, especially in patients over 50 years old, with osteoporosis, or with a history of trauma. [24] Computed Tomography (CT) scan plays a role in diagnosis to obtain more detailed information on bone structure, especially in already diagnosed fractures or when there are reasonable doubts. It is also useful when a Magnetic Resonance Imaging (MRI) cannot be performed. Its advantage is the speed in obtaining images and the ability to study bone structure, as well as traumatic visceral pathologies, with the main drawback being the exposure to ionizing radiation. [24, 26] CT scan is especially useful in polytraumatized patients. MRI has proven to be a very useful test due to its non-invasive nature and high resolution on soft tissues such as intervertebral discs, ligaments, spinal cord, perivertebral tissues… That is the reason why MRI is the most commonly used technique for studying low back and radicular pain, as well as for diagnosing disc herniations and characterizing them. [24] Other techniques such as myelography, myelo-CT, or nuclear medicine tests with radioisotopes are less commonly used in clinical practice. [24] The use of electromyography is unclear. It can be useful to confirm the presence of other neurological disease or in cases with neurological symptoms. [26].

It is important to remember that there is a significant clinical-radiological dissociation, with a significant percentage of asymptomatic individuals having lumbar disc structure alterations, reaching up to 30% of disc pathology in 30-year-olds and up to 84% in 80-year-olds. [27] Although disc extrusion or severe nerve compression is associated with ipsilateral leg pain, other MR findings such as mild to moderate compression, or disc degeneration do not correlate with symptomatic disease. [27].

## Intervention Strategies

The treatment of this pathology can be conservative, surgical, or pharmacological, as detailed below.

### Conservative Treatment

The scientific evidence determines that conservative treatment is ideal in the initial phase of LBP to avoid the risks and possible complications of surgery. [28–30] Currently, the conservative treatment most supported by the literature is based on physiotherapy using multimodal approaches based on therapeutic education, manual therapy (MT), and therapeutic exercise (TE). [31].

Therapeutic education on the neurophysiological mechanisms of pain is a tool that aims to change maladaptive beliefs and erroneous thoughts that interfere with the perception of pain and lead to an increase in the presence of psychological variables such as fear of movement, hypervigilance and catastrophism, thus increasing the perpetuation and chronicity of pain. [32] This technique is considered a key element in the multimodal approach to these patients. The ideal model of education used in these patients should advocate empowering them to take an active role in their recovery. [33, 34] Studies in chronic LBP conditions have shown benefits when measuring disability, catastrophizing, anxiety, self-efficacy, performance and fitness. [35–37].

Within this approach, the use of different MT techniques based on mobilisation and manipulation can bring relief in the short term, improving function and quality of life. Techniques such as Mulligan and neural mobilisation have been shown to significantly reduce radicular pain in patients diagnosed with symptomatic lumbar hernia, as well as improving the range of motion of the lumbar spine. [38–40] In this line, a recent study shows that in subjects with lumbar hernia and long-standing radicular pain, the application of McKenzie in combination with education led to a significant improvement in variables such as quality of life, pain intensity and perceived degree of disability. [41].

The performance of MT in this population underlies the generation of a window of analgesia that could be used by the physiotherapist and the patient for the execution of progressive exercise. It should be noted that the effects of MT are immediate or short term and can be neurophysiological and/or mechanical in nature. In addition to the techniques mentioned above, the application of traction has proved beneficial in subjects with lumbar hernia and radicular component, but these effects cannot be attributed solely to the mechanical component, as there are few in vivo studies in humans. [42–45] In this line, a 2017 systematic review concludes that the application of MT and lumbar traction may have an impact on disc physiology, but more research studies are needed to support this argument. [46] Therefore, we can highlight that currently the use of various MT techniques are more supported by their neurophysiological effects at the central level, generating either medullary changes, decreasing activity at the level of the posterior horn, or supramedullary changes, decreasing the activation of regions such as periaqueductal grey matter, the amygdala, the anterior cingulate cortex, and the rostral ventromedial medulla. [47–49] On the other hand, it has been studied that at the peripheral level, MT can cause changes directly on inflammatory mediators and peripheral nociceptors. [50] In addition, sympathoexcitatory autonomic responses and an opioid response occur in association. [47–51] Indirectly, variables such as placebo, expectations and psychosocial factors may play a role in the mechanisms of MT. [51].

Certainly, the central point in this multimodal approach is based on therapeutic exercise (TE). There is an inverse association between the level of physical activity and the intensity of pain and disability in subjects with recurrent or chronic LBP. [52] A low level of physical activity can lead to a reduction in neuromuscular efficiency and a decrease in strength, causing a series of negative consequences on functionality and postural control. [53, 54].

In many cases, patients with symptomatic lumbar herniated discs have functional instability, so scientific evidence suggests that the ideal is to combine different exercise modalities, such as motor control exercise targeting stabilizing muscles (*i.e.*, transverse abdominis, multifidus and pelvic floor), together with a strength exercise program of the abdominal wall and superficial lumbar region that will help reduce the load on the spine. [55, 56].

The clinical effects of exercise in patients diagnosed with symptomatic lumbar hernia have been extensively studied. However, the neurophysiological mechanisms underlying the medium- to long-term benefits of therapeutic exercise have not yet been identified. Therapeutic exercise has demonstrated benefits on variables such as pain intensity, disability, sleep quality, mobility, quality of life, mental health, balance, strength, and stability in patients with symptomatic lumbar hernia. [57–61] Regarding the biological effects of exercise on this clinical condition, scientific evidence determines that the three fundamental pillars are the reduction of nerve compression, the release of inflammatory mediators and the role of the immune system in pain modulation. [62–65].

On the other hand**,** there are techniques that can be used between conservative interventions and surgery, such as epidural corticosteroid infiltrations or lumbar transforaminal injection of steroids, which are useful tools in patients with contained disc herniations without neurological deficit and can avoid surgery. [66, 67].

### Surgical Treatment

Surgical treatment option is only recommended when the patient's condition is severe, does not improve with conservative treatment, or presents neurological symptoms such as anaesthesia, incontinence, sudden paresis of an extremity, or cauda equina syndrome. [68, 69] Besides, two of the controversies around herniated disc surgery are the followings: [1] when is the best time to perform the surgery? and [2] what is the best surgical technique available?

Regarding the timing for surgery, whether neurological symptoms are not detected, early surgery management only has benefits in the first weeks, with quicker return to activities and less leg pain. In contrast, with prolonged conservative treatment, although the recovery is slower in the first weeks, it is estimated that only around half of patients need to undergo a disc herniation surgery. [70] Considering that around 60–70% of lumbar disc herniations reabsorb within approximately a year and that, regardless of the treatment used, it is estimated that 46.2% of patients with lumbar disc herniation experience LBP at some point during long-term follow-up, decision-making is not always homogeneous among spine surgeons. [71, 72] When cost-effectiveness is analysed, it seems that early surgery is more favourable than conservative treatment in a 4-year follow-up, especially due to indirect costs. [73].

Surgical approaches for a lumbar disc herniation include open discectomy, microdiscectomy, endoscopic microdiscectomy, tubular discectomy, or percutaneous discectomy. Several trials comparing different techniques have been done, with heterogeneous results. [66, 74] Although open discectomy and microdiscectomy remain as the gold standard surgical treatments, for some years now, it seems that endoscopy has similar clinical outcomes with a lower rate of complications. [66] Two main techniques have been described for an endoscopic approach: unilateral biportal endoscopic discectomy, and percutaneous endoscopic lumbar discectomy. Both of them have shown similar clinical outcomes in comparison to microdiscectomy or open discectomy, while cost-effectiveness studies have shown better results for endoscopic techniques. [75, 76] When comparing both endoscopic techniques, safety and efficacy are similar, although unilateral biportal endoscopic discectomy seems to have a lower rate of recurrence, and higher dural sac area. [77] No cost-effectiveness studies have been done between endoscopic techniques, but shorter surgical times, less bleeding and shorter hospital stays were described with percutaneous endoscopic lumbar discectomy. [77].

Lastly, other percutaneous approaches such as percutaneous electrothermal nucleoplasty, intradiscal ozone injection or intradiscal platelet rich plasma injections have shown little evidence. [78].

### Pharmacological Treatment

In terms of medication use, the first line of action is usually conservative, with the prescription of paracetamol and anti-inflammatory drugs used primarily for pain relief. [79] In addition, drugs such as opioid analgesics, antidepressants, muscle relaxants, cytokine inhibitors, or even epidural injections of corticosteroids are also used. However, current research indicates a paucity of high-quality evidence supporting the efficacy of pharmacological treatments for patients with chronic pain resulting from a clinical picture of lumbar disc herniation. Nonsteroidal anti-inflammatory drugs and cytokine inhibitors have not demonstrated significant improvements in mitigating the symptoms of sciatica resulting from a herniated disc when the patient is in a state of symptom maintenance and recurrence. [79, 80].

According to the North American Spine Society, there is insufficient evidence to support the recommendation of the use of IV glucocorticosteroids, 5-hydroxytryptamine receptor inhibitors, gabapentin, agmatine sulfate, or amitriptyline. [78] Furthermore, tumor necrosis factor-alpha inhibitors do not seem to provide a benefit in the treatment of lumbar disc herniation with radiculopathy. [78].

In addition, there are publications in the scientific literature that study the potential effect of new possible treatments such as nutraceutical supplements in combination with Oxygen-Ozone Therapy, or even the epidural injection of platelet-rich plasma. [81, 82] However, these studies are usually limited, with small sample sizes, so their results should be taken with caution.

In short, the approach most consistent with the evidence in the scientific literature is the conservative intervention of physiotherapy using multimodal approaches based on therapeutic education, manual therapy and therapeutic exercise [31], as previously described. Moreover, in the context of acute and subacute pain, this physiotherapeutic triad, in combination with paracetamol and anti-inflammatory drugs may be of clinical benefit to the sufferer. (79).

## Conclusion

Clinical practice guidelines determine that the most appropriate intervention for people suffering from LBP is hitherto a conservative treatment based on therapeutic exercise, manual therapy and education, followed by surgical intervention if the criteria are met. Consequently, it is essential to continue research into the treatment of herniated discs, not only to continue improving the symptoms and quality of life of the patient, but also to seek therapies that improve, reduce or eliminate the pathology itself.

## Data Availability

No datasets were generated or analysed during the current study.

## Declarations

### Permission to Reproduce Material from other Sources

Figures are originals, they have not been acquired from other studies.
